# Traditional and Novel Adiposity Indicators and Pancreatic Cancer Risk: Findings from the UK Women’s Cohort Study

**DOI:** 10.3390/cancers13051036

**Published:** 2021-03-02

**Authors:** Sangeetha Shyam, Darren Greenwood, Chun-Wai Mai, Seok Shin Tan, Barakatun Nisak Mohd Yusof, Foong Ming Moy, Janet Cade

**Affiliations:** 1 Division of Nutrition and Dietetics, School of Health Sciences, International Medical University (IMU), Kuala Lumpur 57000, Malaysia; Sangeethashyam@imu.edu.my (S.S.); SeokShin_Tan@imu.edu.my (S.S.T.); 2 Centre for Translational Research, IMU Institute for Research, Development and Innovation (IRDI), Kuala Lumpur 57000, Malaysia; 3 School of Medicine, University of Leeds, Leeds LS2 9LN, UK; D.C.Greenwood@leeds.ac.uk; 4 Leeds Institute for Data Analytics, University of Leeds, Leeds LS2 9LN, UK; 5 Centre for Cancer and Stem Cells Research, Institute for Research, Development and Innovation (IRDI), International Medical University, Kuala Lumpur 57000, Malaysia; chunwai_mai@imu.edu.my; 6 State Key Laboratory of Oncogenes and Related Genes, Renji-Med X Clinical Stem Cell Research Center, Department of Urology, Ren Ji Hospital, School of Medicine, Shanghai Jiao Tong University, Shanghai 200127, China; 7 Department of Nutrition and Dietetics, Faculty of Medicine and Health Sciences, Universiti Putra Malaysia, Serdang 43400, Malaysia; bnisak@upm.edu.my; 8 Department of Social & Preventive Medicine, Faculty of Medicine, University of Malaya, Kuala Lumpur 50603, Malaysia; moyfm@ummc.edu.my; 9 Nutritional Epidemiology Group, School of Food Science and Nutrition, University of Leeds, Leeds LS2 9LN, UK

**Keywords:** obesity, pancreatic cancer, UKWCS, clothing sizes, women

## Abstract

**Simple Summary:**

Pancreatic cancer has a poor survival rate and its modifiable risks are poorly understood. We investigated the association between both traditional (BMI, waist and hip circumference and waist–hip ratio) and novel (standard UK clothing sizes) adiposity indicators as predictors of pancreatic cancer risk among women enrolled in the UK women’s cohort study (UKWCS). When adjusted for known confounders like age, education, smoking and physical activity, hip circumference and skirt size were both significant predictors of pancreatic cancer risk in the median follow-up of approximately 19 years. BMI became a significant predictor of pancreatic cancer risk when potential latent cases of pancreatic cancer were excluded from the analysis. Thus, adiposity indicators, specifically hip circumference and standard skirt size, are useful to predict pancreatic cancer among women and should therefore be routinely documented in both national surveys and epidemiological studies.

**Abstract:**

(1) Background: We studied the association of both conventional (BMI, waist and hip circumference and waist–hip ratio) and novel (UK clothing sizes) obesity indices with pancreatic cancer risk in the UK women’s cohort study (UKWCS). (2) Methods: The UKWCS recruited 35,792 women from England, Wales and Scotland from 1995 to 1998. Cancer diagnosis and death information were obtained from the National Health Service (NHS) Central Register. Cox’s proportional hazards regression was used to evaluate the association between baseline obesity indicators and pancreatic cancer risk. (3) Results: This analysis included 35,364 participants with a median follow-up of 19.3 years. During the 654,566 person-years follow up, there were 136 incident pancreatic cancer cases. After adjustments for age, smoking, education and physical activity, each centimetre increase in hip circumference (HR: 1.03, 95% CI: 1.01–1.05, *p* = 0.009) and each size increase in skirt size (HR: 1.12, 95% CI: 1.02–1.23, *p* = 0.041) at baseline increased pancreatic cancer risk. Baseline BMI became a significant predictor of pancreatic cancer risk (HR: 1.04, 95% CI: 1.00–1.08, *p* = 0.050) when latent pancreatic cancer cases were removed. Only baseline hip circumference was associated with pancreatic cancer risk (HR: 1.03, 95% CI: 1.00–1.05, *p* = 0.017) when participants with diabetes at baseline were excluded to control for reverse causality. (4) Conclusion: Hip circumference and skirt size were significant predictors of pancreatic cancer risk in the primary analysis. Thus, hip circumference is useful to assess body shape relationships. Additionally, standard skirt sizes offer an economical and objective alternative to conventional obesity indices for evaluating pancreatic cancer risk in women.

## 1. Introduction

Pancreatic cancer has one of the poorest 5-year survival statistics, especially if diagnosed late [1,2,3]. While the survival data point to the importance of preventative strategies and early detection, pancreatic cancer continues to be an understudied area in oncology, and the understanding of its aetiological factors is nascent in comparison to other cancers [4]. Obesity, specifically visceral obesity, is among the few known modifiable risk factors associated with pancreatic cancer [5,6,7,8,9,10,11,12]. Obesity is hypothesised to increase pancreatic cancer risk through mechanistic pathways that include altered adipokine secretion, insulin resistance, inflammation, oxidative stress and the bioavailability of sex hormones [13].

Conventional adiposity indicators, namely weight, body mass index (BMI), waist circumference and waist-to-hip ratio, are routinely used in research and in practice to identify the risk of chronic diseases. These indicators require trained personnel to perform the measurements, and thus, from a public health perspective, they are labour-intensive. Therefore, for population studies or community screening purposes, self-reported standard clothing sizes are an objective, easy-to-measure alternative adiposity indicator and can be reliably reported by respondents [14,15,16,17,18].

Clothing sizes have been found useful to predict the risk of various cancers in both men and women. Among a sub-cohort of weight-stable women from the Netherlands Cohort Study, self-reported Dutch skirt size data significantly predicted the risk of endometrial cancer in women [14]. In the same cohort, trouser size was also strongly positively associated with cancer of the distal colon among men, although clothing sizes showed no clear trend for colon cancer risk among women [15]. In the United Kingdom, endometrial cancer risk in postmenopausal women was associated with their clothing size at the age of 20 years [17]. In the UK Collaborative Trial of Ovarian Cancer Screening (UKCTOCS), the skirt size at study enrolment was associated with postmenopausal breast cancer risk, independent of BMI [18]. More recently, among the postmenopausal women enrolled in the UKWCS, self-reported UK blouse and skirt sizes were positively associated with breast cancer risk [16]. There are no available data to evaluate the use of clothing size to predict the risk of pancreatic cancer.

Given the lack of research in relation to risk factors for pancreatic cancer worldwide, there is a need for more data to gauge the magnitude of association between pancreatic cancer risk and obesity indicators. Most studies are too small or short-term to be able to explore the risk factors associated with pancreatic cancer. Furthermore, the low incidence rate of pancreatic cancer (United Kingdom = 6.3 per 100,000 age-standardised incidence in women (3)) requires inevitably large and long-term studies to understand these associations. The UKWCS, with its sufficiently large enrolment and long follow-up, is one of a few studies which can be used to explore these associations. Thus, we explored the relationships between pancreatic cancer risk and both conventional (BMI, waist circumference, hip circumference) and novel (skirt and blouse sizes) adipose indicators using the UK Women’s Cohort Study (UKWCS) data.

## 2. Materials and Methods

### 2.1. The UKWCS Data

#### 2.1.1. Participants

The recruitment and characteristics of the UKWCS participants have been described previously [19]. Briefly, the UKWCS recruited a total of 35,792 women between 1995 and 1998. They were aged 35–69 years at baseline and lived in England, Wales and Scotland. A total of 35,372 women returned the baseline postal questionnaire.

#### 2.1.2. Pancreatic Cancer Case Definition and Ascertainment

Subjects were flagged with the NHS Central Register for both cancer diagnosis and death notification. Incident cancers and cause of death were coded according to the International Classification of Diseases 9 and 10. Pancreatic cancer incidence data were obtained from the linked data from the NHS Central Register for both cancer diagnosis and death notification.

#### 2.1.3. Assessment of Adipose Indicators

Anthropometric measures at baseline, including weight, height, waist and hip circumference, were self-recorded at the time of enrolment. BMI and waist–hip ratio were calculated. BMI was categorised into the four groups of underweight, normal weight, overweight and obese according to the WHO criteria. Skirt and blouse sizes, according to UK clothing standards, were reported by the participants at baseline, with the clothing sizes recorded using even numbers between 6 and 28.

### 2.2. Statistical Analysis

Pearson correlation coefficients between adiposity measures were calculated. The association between obesity and pancreatic cancer risk within the UKWCS was explored using Cox’s proportional hazards regression. Linear trends were also studied using the adiposity indices as continuous variables. Confounding variables to be included in the analysis were identified using a directed acyclic graph (DAG). These variables were age, education, smoking status and hours spent on vigorous physical activity per day, measured at the time of participant enrolment.

A preliminary simple model presented the age-adjusted hazard ratio (HR) for the adiposity indicators. A second fully adjusted model controlled for age, smoking status, education and physical activity. Two additional sensitivity analyses were carried out to rule out reverse causality: (i) excluding all participants who were censored or were diagnosed with pancreatic cancer within 3 years of enrolment, to account for latent pancreatic cancer cases at baseline; and (ii) excluding all participants with diabetes at baseline, to account for potential reverse causality.

All statistical analyses were conducted using STATA version 15 (StataCorp. 2017. Stata Statistical Software: Release 15. College Station, TX, USA: StataCorp LLC.).

### 2.3. Institutional Approvals and Data Access

The UKWCS has ethical approval as a research database (REC reference: 17/YH/0144) with a Public Health England data sharing contract (ODR1718_148). The UKWCS data are held securely on the Integrated Research Campus of the University of Leeds. The data were accessed through a virtual research environment using a token and handled as per the requirements of the European Union General Data Protection Regulation (GDPR). Institutional approvals were also obtained from the University of Leeds and the International Medical University (IMU) before the start of the project.

## 3. Results

### 3.1. Demographic Characteristics of the Participants at Enrolment

After excluding the prevalent cases of pancreatic cancer (*n* = 15) at baseline, the total number of participants available for this analysis was 35,364, with a median follow-up of 19.3 years. In the follow-up of 654,566 person-years, there were 136 incidents of pancreatic cancer cases, resulting in an incidence rate of pancreatic cancer in the UKWCS of 0.21 per 1000 person-years.

The mean (SD) age of the participants at enrolment was 52 (9) years, the majority of the participants (*n* = 29,847, 84%) had an education above O-Level and 17,781 (53%) of the women were postmenopausal. At enrolment, 3810 (11%) of the participants were smokers, 9593 (28%) reported being on a vegetarian diet and 441 (1%) reported being vegan. Subjects’ self-reported participation in vigorous activity was highly variable and ranged from 0 to 14 h/day, with a mean (SD) of approximately 15 (29) minutes per day. Two percent (*n* = 644) of the participants reported having diabetes at baseline. Descriptive characteristics of participants at baseline data collection are summarised by pancreatic cancer incidence in Table 1.

### 3.2. Anthropometric Characteristics of the Participants at Enrolment

Anthropometric details of participants at baseline data collection are summarised by pancreatic cancer incidence in Table 1. The mean (SD) BMI at enrolment was 24.5 (4.3) kg/m^2^. The mean (SD) waist and hip circumference in centimetres were 73.5 (9.3) and 98.6 (8.4), respectively, and the waist–hip ratio was 0.75 (0.06). At enrolment, the reported blouse and skirt sizes ranged between 6 and 28.

Pearson’s correlation coefficients between conventional anthropometric measures and UK clothing sizes are shown in Table 2. BMI and waist and hip circumferences were highly correlated in this cohort of women. Skirt size showed a higher degree of correlation with both general (BMI) and central obesity indicators (waist and hip circumference) than blouse size.

### 3.3. Adiposity and Pancreatic Cancer Risk

Among the conventional adiposity indicators, a trend for higher pancreatic cancer incidence with increasing baseline BMI and increasing waist and hip circumference was observed, across the length of the follow-up (Table 2). Women who were obese, with a BMI (≥30.0 kg/m^2^) at recruitment, had the highest incidence rates of pancreatic cancer. In the simple model adjusted only for age, when compared to normal-weight individuals, individuals with obesity had a 72% higher risk of pancreatic cancer (HR: 1.72, 95% CI: 1.04 −2.84, *p* = 0. 033). However, in the fully adjusted model that was adjusted for age, education, smoking status and physical activity, the increased cancer risk with higher BMI was no longer statistically significant. In the fully adjusted model, only hip circumference remained significantly associated with pancreatic cancer risk (Table 3). There was an increase of 3% in risk for every centimetre increase in hip circumference, after adjustment for confounders (HR: 1.03, 95% CI: 1.01 to 1.05, *p* = 0.009). Waist circumference and waist–hip ratio showed no significant associations with pancreatic cancer incidence in either model (Supplementary Figure S1).

Self-reported clothing sizes of the participants at baseline were also found to be associated with pancreatic cancer incidence over the duration of the study. The incidence of pancreatic cancer was highest among women who reported wearing UK blouse or skirt sizes ≥20 at enrolment. In the fully adjusted models, each size increase in blouse and skirt sizes (e.g., from size 12 to size 14) increased the HR of pancreatic cancer by 8% (HR: 1.08, 95% CI: 0.98 to 1.21) and 12% (HR: 1.12, 95% CI: 1.02 to 1.23), respectively (Supplementary Figure S2).

After the exclusion of pancreatic cancer cases reported within 3 years of recruitment, the association between BMI and pancreatic cancer risk became statistically significant (HR: 1.04, 95% CI: 1.00 to 1.08). However, the results of this sensitivity analysis did not greatly affect the magnitude of these associations. A second sensitivity analysis that excluded participants with diabetes at baseline also did not change the magnitude of these associations between obesity indices and pancreatic cancer. Nevertheless, the analysis attenuated the statistical significance of all these associations. Both sensitivity analyses also revealed the non-linear nature of the association of BMI with the risk of pancreatic cancer, which was not observed in the primary analysis. The risk of pancreatic cancer was higher among the underweight as well as the overweight and obese categories when compared to individuals in the normal-weight category (Supplementary Tables S1 and S2).

## 4. Discussion

In the current analysis, we investigated the associations between conventional adiposity indicators (BMI, waist circumference, hip circumference and waist–hip ratio) and pancreatic cancer risk among the women enrolled in the UKWCS. We also investigated UK clothing sizes as proxy measures for the conventional adiposity indices and evaluated their association with pancreatic cancer risk. In the primary analysis, while BMI and waist and hip circumference showed trends for positive associations with pancreatic cancer risk, in the fully adjusted model, only hip circumference remained a significant predictor of pancreatic cancer risk. The association between BMI and pancreatic cancer risk became statistically significant in the sensitivity analysis that removed potential latent cases of pancreatic cancer at baseline. This was expected given that weight loss precedes the development of other symptoms of pancreatic cancer [20].

The significant association between hip circumference and pancreatic cancer seen in the primary analysis remained unaltered in the sensitivity analyses. These findings are interesting in the context of waist circumference and waist–hip ratio being widely viewed as more effective indicators of central obesity-related health risks compared to hip circumference [21]. The lack of association between either waist circumference or the waist–hip ratio and pancreatic cancer risk observed in the UKWCS is in line with findings from the French E3N longitudinal prospective cohort [22]. Similar to the observations in the current analysis, waist circumference and waist–hip ratio were not significantly associated with breast cancer risk in the French E3N longitudinal prospective cohort. However, hip circumference was associated with the risk of breast cancer irrespective of waist circumference in premenopausal, but not postmenopausal, women in this French cohort [22]. Both the UKWCS and the French E3N cohort study collected self-reported body weight and circumference measurements among women. The findings from these studies point to the differential effects of the location of fat storage and body shape on cancer risk and the ability of self-reported body weight and size measurements to appropriately capture these risks. These findings also suggest that hip circumference should continue to be assessed in evaluating obesity-related disease risk in population studies.

The highest incidence of pancreatic cancer in the UKWCS occurred in women with UK blouse or skirt sizes ≥20. However, increases in skirt size were more significantly associated with pancreatic cancer risk compared to similar increases in blouse size. The magnitude of the association between skirt size and pancreatic cancer risk is in the range of the previously reported estimates for other cancers [14,16]. Earlier reports from the UKWCS that included only postmenopausal women showed that, in fully adjusted linear models, each size increase in skirt size was associated with a 14% (95% CI: 6–22%, *p* < 0.001) increase in breast cancer risk over the course of 17 years [16]. Previously, findings from the UKCTOCS showed that each skirt size increase at study enrolment was associated with a 5% (95% CI: 1% to 9%, *p* = 0.006) increase in postmenopausal breast cancer risk in the fully adjusted model, over a median 4-year follow-up of older women [18]. Similarly, among the participants enrolled in the Netherlands Cohort Study, an 11% (95% CI: −7% to 32%, *p* = 0.06) increase in relative risk (RR) for endometrial cancer was reported for each size increase in skirt size (used as a continuous variable) during a 13-year follow-up [14]. In the current analysis that included all the women enrolled in the UKWCS, irrespective of their menopausal status, the fully adjusted model showed a 12% (95% CI: 2 to 23%, *p* = 0.041) increase in pancreatic cancer risk over a median follow-up of 19 years when the self-reported UK skirt size at baseline went up a size.

In the UKWCS, a higher skirt size, but not blouse size, was associated with a significantly increased risk of pancreatic cancer. This could be because blouse size is more affected by upper-body fat distribution, while skirt size considers both waist and hip measurements and therefore is more reflective of visceral obesity. This argument is supported by the higher degree of correlation observed in this study for skirt rather than blouse size with traditional adiposity indices. Several studies have implicated insulin resistance and the subsequent chronic pancreatic inflammation associated with visceral obesity in the pathogenesis of pancreatic cancer [5,6,7,8,9,10,11,12]. When a sensitivity analysis was performed by excluding those with diabetes at baseline, the association of all adiposity indicators with pancreatic cancer risk was attenuated. This suggests that the effect of obesity on increasing pancreatic cancer risk could be at least partially moderated through adiposity-related insulin resistance and alterations in glucose homeostasis [13].

The strengths of using the UKWCS data for the current analysis include the prospective design, the involvement of a large single-gender population, and the sufficiently long follow-up period to account for the incidence of pancreatic cancer. Moreover, the UKWCS collected data on weight, waist and hip circumference and UK clothing sizes for blouse and skirt., thus facilitating the evaluation of both measures.

We also acknowledge the following limitations. Due to the rare nature of the disease, pancreatic cancer cases were limited even within this large cohort, potentially reducing the power to evaluate the associations of interest. Post-hoc power calculations showed that the current analysis of both hip circumference and skirt size with pancreatic cancer incidence yielded powers of 77% and 67%, respectively. The limited number of cases also considerably increased the probability of committing a type II error when evaluating the association between BMI and pancreatic cancer. Hence, these results must be interpreted with caution. However, it should be noted that there is a consistency in the magnitude of associations between all adiposity indicators studied and pancreatic cancer incidence, suggesting that the point estimates may be close to the actual values. Secondly, this study used self-reported measures and clothing sizes. Self-reported body weight and measurements are likely to be misreported due to social desirability bias [23,24]. However, the validity of such measures in large epidemiological studies, specifically in European cohorts, has been documented [25,26]. Moreover, clothing sizes have shown a high degree of correlation with professionally measured waist circumference in adult men and women [27], thereby making them alternative markers for adiposity [28].

Furthermore, changes in body weight, shape and health behaviour throughout the study were not factored in the modelling. Since weight loss is a symptom and consequence of pancreatic cancer [20], such an adjustment may attenuate any existing association between obesity and pancreatic cancer. Finally, clothing sizes may also be influenced by individual preferences for fit rather than bodyweight status. However, as described earlier, there is evidence for the use of clothing sizes as strong surrogates for obesity, central obesity and body shape [14,29]. This study adds to existent evidence that demonstrates moderate to high levels of correlation between self-reported BMI and standard clothing sizes in women [17,18]. Thus, the current study provides further evidence to attest to the usefulness of collecting clothing size information in epidemiological studies and national surveys, where readymade standard-sized clothing is commonly used. Clothing sizes are simple, rapid, economical and non-invasive proxy measures for the conventional adiposity indices. Finally, this study did not account for the types of pancreatic cancers in the cohort. Given the rarity of these cancers, it was decided that further categorising the pancreatic cancers would reduce the power to detect any association.

## 5. Conclusions

Pancreatic cancer incidence increased with increases in conventionally used obesity indices (BMI, waist and hip circumference and waist–hip ratio). In our primary analysis, only hip circumference remained a significant predictor of cancer risk when adjusted for known confounders. Hip circumference measurements should be included in population studies to further assess the effects of fat storage location and body shape in determining disease risks. When the analysis excluded potential latent pancreatic cancer cases at baseline, BMI was also significantly positively associated with pancreatic cancer risk. The risk for pancreatic cancer increased with each size increase in skirt size. Therefore, when standardised clothing sizes are widely used in a population, skirt sizes offer an objective, economical and robust alternative to traditional obesity indices used to evaluate pancreatic cancer risk in women. Thus, this study contributes further evidence to the utility of self-reported clothing size as an indicator of obesity-related disease risk among women and makes a case for its routine documentation in epidemiological studies and national surveys.

## Figures and Tables

**Table 1:** Descriptive characteristics at enrolment in the UK women’s cohort study (UKWCS) by pancreatic cancer incidence.

Enrolment Characteristic	Pancreatic Cancer Cases (*n* = 136)	Others (*n* = 35,228)
Age (y) Mean (SD)	59 (8)	52 (9)
Formal Education (above O-Level) *n* (%)	104 (76%)	29,743 (84%)
Health Behaviours and Diabetes		
Vegetarian *n* (%)	27 (21%)	9566 (28%)
Vegan *n* (%)	4 (3%)	437(1%)
Walking h/day Mean (SD)	0.89 (0.70)	0.93 (0.93)
Vigorous activity h/day Mean (SD)	0.21 (0.41)	0.25 (0.48)
Prevalence of smoking *n* (%)	21 (15%)	3789 (11%)
Prevalence of diabetes *n* (%)	9 (7%)	637 (2%)
Menopausal status *n* (%)		
Premenopausal	18 (14%)	15,933 (47%)
Postmenopausal	109 (86%)	17,672 (53%)
Anthropometric indices		
BMI (kg/m^2^) Mean (SD)	25 (4)	24 (4)
BMI categories *n* (%)		
Underweight (<18.5 kg/m^2^)	2 (2%)	736 (2%)
Normal weight (18.5–24.9 kg/m^2^)	67 (52%)	21,233 (63%)
Overweight (25.0–29.9 kg/m^2^)	41 (32%)	8585 (25%)
Obese (≥30.0 kg/m^2^)	20 (15%)	3329 (10%)
Waist (cm) Mean (SD)	76.7 (9.8)	73.5 (9.3)
Hip (cm) Mean (SD)	101.9 (8.2)	98.6 (8.4)
Waist–Hip ratio Mean (SD)	0.75 (0.06)	0.75 (0.06)
UK clothing sizes		
Blouse size Mean (SD)	16 (3)	14 (3)
Blouse sizes *n* (%)		
≤10	15 (11%)	5266 (15%)
12	25 (18%)	9726 (28%)
14	36 (26%)	9326 (27%)
16	25 (18%)	5870 (17%)
18	18 (13%)	2624 (8%)
≥20	17 (13%)	2095 (6%)
Skirt size Mean (SD)	16 (4)	14 (3)
Skirt sizes *n* (%)		
≤10	7 (5%)	3529 (10%)
12	21 (16%)	8323 (24%)
14	30 (22%)	9854 (28%)
16	39 (29%)	6967 (20%)
18	12 (9%)	3383 (10%)
≥20	25 (19%)	2670 (8%)

**Table 2:** Correlation between conventional adiposity indices and clothing sizes in the UKWCS.

	BMI	Waist (cm)	Hip (cm)	Skirt Size
Waist (cm)	0.7006			
Hip (cm)	0.7613	0.7665		
Skirt size	0.6241	0.6003	0.6226	
Blouse size	0.3897	0.3762	0.3742	0.5815

**Table 3:** Adiposity indices and hazard ratio (HR) (95% CI) for pancreatic cancer in the UKWCS.

Adiposity Indicator	Person Years	Incidence Rates (per 1000)	Age-Adjusted Model HR (95% CI)	*p* Value	Fully Adjusted Model ^1^ HR (95% CI)	*p* Value
Table. *Cont.* BMI groups at enrolment						
Cases/total		130/34,005	130/33,649		123/31,944	
Underweight	13,471	0.15 (0.04–0.59)	0.98 (0.24–4.00)		0.99 (0.24–4.03)	
Normal weight	398,866	0.17 (0.13–0.21)	1 (Reference)		1 (Reference)	
Overweight	157,513	0.26 (0.19–0.35)	1.29 (0.88–1.91)		1.30 (0.87–1.94)	
Obese	60,049	0.33 (0.21–0.52)	1.72 (1.04–2.84)		1.62 (0.96–2.74)	
Linear (per kg/m^2^)	629,899		1.04 (1.00–1.08)	0.055	1.03 (1.00–1.07)	0.081
Waist circumference (cm)						
Cases/total			116/27,607		109/26,290	
Linear (per cm)			1.02 (1.00–1.03)	0.082	1.02 (1.00–1.03)	0.105
Hip circumference (cm)						
Cases/total			118/27,928		111/26,589	
Linear (per cm)			1.03 (1.01–1.07)	0.008	1.03 (1.01–1.05)	0.009
Waist–Hip Ratio						
Cases/total			115/27,102		108/25,827	
Linear (per unit)			0.64 (0.03–12.28)	0.766	0.49 (0.02–10.62)	0.652
Blouse size						
Cases/total			136/34,463		128/32,830	
≤10	99,504	0.15 (0.09–0.25)	1 (Reference)		1 (Reference)	
12	184,427	0.14 (0.09–0.20)	0.87 (0.46–1.65)		0.81 (0.42–1.54)	
14	173,520	0.21 (0.15–0.29)	1.11 (0.61–2.04)		1.12 (0.61–2.05)	
16	107,627	0.23 (0.16–0.34)	1.06 (0.56–2.02)		1.03 (0.53–1.97)	
18	47,029	0.38 (0.24–0.61)	1.60 (0.80–3.21)		1.46 (0.71–2.97)	
≥20	36,614	0.46 (0.29–0.75)	2.07 (1.03–4.17)		1.73 (0.83–2.62)	
Linear (for each size up)	648,722		1.12 (1.02–1.25)	0.024	1.08 (0.98–1.21)	0.114
Skirt size						
Cases/total			134/34,462		128/32,668	
≤10	67,434	0.10 (0.05–0.22)	1 (Reference)		1 (Reference)	
12	158,247	0.13 (0.09–0.20)	1.09 (0.46–2.57)		1.06 (0.45–2.52)	
14	184,615	0.16 (0.11–0.23)	1.08 (0.47–2.47)		1.07 (0.46–2.45)	
16	127,799	0.31 (0.22–0.42)	1.71 (0.75–3.87)		1.67 (0.73–3.80)	
18	60,806	0.20 (0.11–0.35)	0.98 (0.38–2.53)		0.85 (0.32–2.28)	
≥20	46,708	0.54 (0.36–0.79)	2.75 (1.17–6.47)		2.63 (1.10–6.25)	
Linear (for each size up)	645,609		1.14 (1.02–1.25)	0.014	1.12 (1.02–1.23)	0.041

Fully adjusted model ^1^: adjusted for age, smoking, education and physical activity level.

## Data Availability

The data presented in this study are available on request from the corresponding author. The data are not publicly available due to data protection and privacy regulations.

## Supplementary Materials

The following are available online at https://www.mdpi.com/2072-6694/13/5/1036/s1, Supplementary Table S1: Adiposity indices and HR (95% CI) for pancreatic cancer in the UKWCS sensitivity analysis excluding latent pancreatic cancer at baseline. Table S2: Adiposity indices and HR (95% CI) for pancreatic cancer in the UKWCS sensitivity analysis excluding cases of diabetes at baseline. Supplementary Figure S1: Fully adjusted HR 95 CI) and crude incidence rates for pancreatic cancer, by anthropometric indices. Supplementary Figure S2: Fully adjusted HR (95% CI) and crude incidence rates for pancreatic cancer, by clothing size.

## References

[1] Mettu N.B., Abbruzzese J.L. (2016). Clinical Insights into the Biology and Treatment of Pancreatic Cancer. J. Oncol. Pract..

[2] Kardosh A., Lichtensztajn D.Y., Gubens M.A., Kunz P.L., Fisher G.A., Clarke C.A. (2018). Long-Term Survivors of Pancreatic Cancer: A California Population-Based Study. Pancreas.

[3] Rawla P., Sunkara T., Gaduputi V. (2019). Epidemiology of Pancreatic Cancer: Global Trends, Etiology and Risk Factors. World J. Oncol..

[4] Bouvier A.-M., Uhry Z., Jooste V., Drouillard A., Remontet L., Launoy G., Leone N., French Network of Cancer Registries (Francim) (2017). Focus on an unusual rise in pancreatic cancer incidence in France. Int. J. Epidemiol..

[5] Arslan A.A., Helzlsouer K.J., Kooperberg C., Shu X.O., Steplowski E., Bueno-de-Mesquita H.B., Fuchs C.S., Gross M.D., Jacobs E.J., Lacroix A.Z. (2010). Anthropometric measures, body mass index, and pancreatic cancer: a pooled analysis from the Pancreatic Cancer Cohort Consortium (PanScan). Arch. Intern. Med..

[6] Freisling H., Arnold M., Soerjomataram I., O’Doherty M.G., Ordonez-Mena J.M., Bamia C., Kampman E., Leitzmann M., Romieu I., Kee F. (2017). Comparison of general obesity and measures of body fat distribution in older adults in relation to cancer risk: Meta-analysis of individual participant data of seven prospective cohorts in Europe. Br. J. Cancer.

[7] Gaujoux S., Torres J., Olson S., Winston C., Gonen M., Brennan M.F., Klimstra D.S., D’Angelica M., DeMatteo R., Fong Y. (2012). Impact of obesity and body fat distribution on survival after pancreaticoduodenectomy for pancreatic adenocarcinoma. Ann. Surg. Oncol..

[8] Kahn S.E., Prigeon R.L., Schwartz R.S., Fujimoto W.Y., Knopp R.H., Brunzell J.D., Porte D. (2001). Obesity, body fat distribution, insulin sensitivity and Islet beta-cell function as explanations for metabolic diversity. J. Nutr..

[9] Pischon T., Nothlings U., Boeing H. (2008). Obesity and cancer. Proc. Nutr. Soc..

[10] Tranchart H., Gaujoux S., Rebours V., Vullierme M.P., Dokmak S., Levy P., Couvelard A., Belghiti J., Sauvanet A. (2012). Preoperative CT scan helps to predict the occurrence of severe pancreatic fistula after pancreaticoduodenectomy. Ann. Surg..

[11] Xiao J., Mazurak V.C., Olobatuyi T.A., Caan B.J., Prado C.M. (2018). Visceral adiposity and cancer survival: a review of imaging studies. Eur. J. Cancer Care (Engl.).

[12] Yamane H., Abe T., Amano H., Hanada K., Minami T., Kobayashi T., Fukuda T., Yonehara S., Nakahara M., Ohdan H. (2018). Visceral Adipose Tissue and Skeletal Muscle Index Distribution Predicts Severe Pancreatic Fistula Development After Pancreaticoduodenectomy. Anticancer Res..

[13] Bao B., Wang Z., Li Y., Kong D., Ali S., Banerjee S., Ahmad A., Sarkar F.H. (2011). The complexities of obesity and diabetes with the development and progression of pancreatic cancer. Biochim. Biophys. Acta (BbA) Rev. Cancer.

[14] Hughes L.A.E., Schouten L.J., Goldbohm R.A., van den Brandt P.A., Weijenberg M.P. (2009). Self-reported Clothing Size as a Proxy Measure for Body Size. Epidemiology.

[15] Hughes L.A.E., Simons C.C.J.M., van den Brandt P.A., Goldbohm R.A., van Engeland M., Weijenberg M.P. (2011). Body Size and Colorectal Cancer Risk After 16.3 Years of Follow-up: An Analysis From the Netherlands Cohort Study. Am. J. Epidemiol..

[16] Moy F.M., Greenwood D.C., Cade J.E. (2018). Associations of clothing size, adiposity and weight change with risk of postmenopausal breast cancer in the UK Women’s Cohort Study (UKWCS). BMJ Open.

[17] Yang T.Y.O., Cairns B.J., Allen N., Sweetland S., Reeves G.K., Beral V., Million Women S. (2012). Postmenopausal endometrial cancer risk and body size in early life and middle age: Prospective cohort study. Br. J. Cancer.

[18] Fourkala E.-O., Burnell M., Cox C., Ryan A., Salter L.C., Gentry-Maharaj A., Parmar M., Jacobs I., Menon U. (2014). Association of skirt size and postmenopausal breast cancer risk in older women: A cohort study within the UK Collaborative Trial of Ovarian Cancer Screening (UKCTOCS). BMJ Open.

[19] Cade J.E., Burley V.J., Alwan N.A., Hutchinson J., Hancock N., Morris M.A., Threapleton D.E., Greenwood D.C. (2015). Cohort profile: The UK Women’s cohort study (UKWCS). Int. J. Epidemiol..

[20] Hart P.A., Kamada P., Rabe K.G., Srinivasan S., Basu A., Aggarwal G., Chari S.T. (2011). Weight loss precedes cancer-specific symptoms in pancreatic cancer-associated diabetes mellitus. Pancreas.

[21] Lissner L., Björkelund C., Heitmann B.L., Seidell J.C., Bengtsson C. (2001). Larger Hip Circumference Independently Predicts Health and Longevity in a Swedish Female Cohort. Obes. Res..

[22] Fagherazzi G., Chabbert-Buffet N., Fabre A., Guillas G., Boutron-Ruault M.C., Mesrine S., Clavel-Chapelon F. (2012). Hip circumference is associated with the risk of premenopausal ER−/PR− breast cancer. Int. J. Obes..

[23] Burke M.A., Carman K.G. (2017). You can be too thin (but not too tall): Social desirability bias in self-reports of weight and height. Econ. Hum. Biol..

[24] Gorber S.C., Tremblay M., Moher D., Gorber B. (2007). A comparison of direct vs. self-report measures for assessing height, weight and body mass index: A systematic review. Obes. Rev..

[25] Bolton-Smith C., Woodward M., Tunstall-Pedoe H., Morrison C. (2000). Accuracy of the estimated prevalence of obesity from self reported height and weight in an adult Scottish population. J. Epidemiol. Community Health.

[26] Spencer E.A., Appleby P.N., Davey G.K., Key T.J. (2007). Validity of self-reported height and weight in 4808 EPIC–Oxford participants. Public Health Nutr..

[27] Battram D.S., Beynon C., He M. (2011). The reliability and validity of using clothing size as a proxy for waist circumference measurement in adults. Appl. Physiol. Nutr. Metab..

[28] Vogt M., Puntschart A., Howald H., Mueller B., Mannhart C., Gfeller-tuescher L., Mullis P., Hoppeler H. (2003). Effects of dietary fat on muscle substrates, metabolism, and performance in athletes. Med. Sci. Sports Exerc..

[29] Han T.S., Gates E., Truscott E., Lean M.E.J. (2005). Clothing size as an indicator of adiposity, ischaemic heart disease and cardiovascular risks. J. Hum. Nutr. Diet..

