# Supplementary material for: Traditional and Novel Adiposity Indicators and Pancreatic Cancer Risk: Findings from the UK Women’s Cohort Study
Source: Cancers (Basel). 2021 Mar 2;13(5):1036. doi: 10.3390/cancers13051036 (PMC7957885; doi:10.3390/cancers13051036)
Supplement: Supplementary file 1 [file cancers-13-01036-s001.pdf]

cancers

MDPI

Supplementary material

Traditional and novel adiposity indicators and pancreatic cancer risk: findings from the UK Women's Cohort Study

Sangeetha Shyam, Darren Greenwood, Mai Chun Wai, Tan Seok Shin, Barakatun Nisak Mohd Yusof, Foong Ming Moy and Janet Cade

Table S1. Adiposity indices and HR (95% CI) for pancreatic cancer in the UKWCS- sensitivity analysis excluding latent pancreatic cancer at baseline.

Adiposity Indicator	Age adjusted model HR (95% CI)	P value	Fully adjusted model[1] HR (95% CI)	P value
**BMI groups at enrolment**				
Cases/total	123/ 33,322		116/ 31,652	
Underweight	1.04 (0.26- 4.27)		1.05 (0.26-4.31)	
Normal weight	1 (Reference)		1 (Reference)	
Overweight	1.28 (0.85-1.92)		1.29 (0.85-1.95)	
Obesity	1.84 (1.11-3.05)		1.73 (1.02-2.94)	
Linear (per kg/m^2)	1.04 (1.00-1.08)	0.032	1.04 (1.00-1.08)	0.050
**Waist circumference (cm)**				
Cases/total	110 /27,339		103/26,047	
Linear (per cm)	1.02 (1.00-1.03)	0.119	1.01 (0.99-1.03)	0.153
**Hip circumference (cm)**				
Cases/total	112/27,647		105/26,336	
Linear (per cm)	1.03 (1.01-1.05)	0.008	1.03 (1.01-1.05)	0.009
**Waist-Hip Ratio**				
Cases/total	109/26,481		102/25,590	
Linear (per unit)	0.40 (0.02-8.81)	0.564	0.30 (0.01-7.30)	0.458
**Blouse size**				
Cases/total	129/34,306		121/32,529	
≤10	1 (Reference)		1 (Reference)	
12	0.86 (0.44-1.66)		0.79(0.40-1.55)	
14	1.20(0.65-2.22)		1.21 (0.65-2.24)	
16	1.05 (0.54-2.06)		1.02 (0.52-2.00)	
18	1.74 (0.86-3.53)		1.58 (0.76-3.27)	
≥ 20	1.99 (0.95-4.14)		1.61 (0.74-3.51)	
Linear (for each size up)	1.12 (1.02-1.25)	0.028	1.08 (0.98-1.21)	0.138
**Skirt size**				
Cases/total	127/34,132		119/32,372	
≤10	1 (Reference)		1 (Reference)	
12	0.99 (0.42-2.36)		0.96 (0.40-2.31)	
14	1.05 (0.46-2.42)		1.04 (0.45-2.41)	
16	1.69 (0.74-3.83)		1.65 (0.72-3.77)	
18	0.92 (0.35-2.40)		0.78 (0.29-2.15)	

≥ 20	2.75 (1.09-6.13)		2.45 (1.02-5.89)	
Linear (for each size up)	1.14 (1.02-1.25)	0.017	1.12 (1.00-1.25)	0.051

Fully adjusted model [1]: adjusted for age, smoking, education and physical activity level. This analysis excluded participants who were censored or were diagnosed with pancreatic cancer within 3 years of enrolment.

Table S2. Adiposity indices and HR (95% CI) for pancreatic cancer in the UKWCS- sensitivity analysis excluding cases of diabetes at baseline.

Adiposity Indicator	Age adjusted model HR (95% CI)	P value	Fully adjusted model[1] HR (95% CI)	P value
**BMI groups at enrolment**				
Cases/total	108/ 30,432		95/ 24,049	
Underweight	1.13 (0.28- 4.64)		1.13 (0.28-4.65)	
Normal weight	1 (Reference)		1 (Reference)	
Overweight	1.23 (0.79-1.89)		1.27 (0.83-1.98)	
Obesity	1.75 (1.01-3.05)		1.87 (1.03-3.17)	
Linear (per kg/m^2)	1.03 (0.99- 1.08)	0.114	1.04 (1.00-1.08)	0.081
**Waist circumference (cm)**				
Cases/total	97 /24,983		95/24,049	
Linear (per cm)	1.02 (1.00-1.04)	0.086	1.02 (1.00-1.04)	0.071
**Hip circumference (cm)**				
Cases/total	98/25,240		96/24,287	
Linear (per cm)	1.03 (1.00-1.05)	0.025	1.03 (1.00-1.05)	0.017
**Waist-Hip Ratio**				
Cases/total	96/24,539		94/23,633	
Linear (per unit)	0.80 (0.03-21.51)	0.896	0.79 (0.03-21.83)	0.891
**Blouse size**				
Cases/total	113/31,281		111/29,979	
≤10	1 (Reference)		1 (Reference)	
12	0.79 (0.41-1.52)		0.77(0.40-1.49)	
14	0.95(0.51-1.78)		0.96 (0.52-1.80)	
16	0.92 (0.47-1.81)		0.89 (0.45-1.75)	
18	1.39 (0.67-2.92)		1.44 (0.69-3.01)	
≥ 20	1.44 (0.65-3.25)		1.46 (0.65-3.27)	
Linear (for each size up)	1.06 (0.94-1.21)	0.281	1.06 (0.94-1.21)	0.267
**Skirt size**				
Cases/total	112/31,130		110/29,838	
≤10	1 (Reference)		1 (Reference)	
12	0.99 (0.41-2.35)		1.01 (0.98-2.42)	
14	1.03 (0.45-2.38)		1.02 (0.44-2.37)	
16	1.46 (0.63-3.36)		1.46 (0.63-3.37)	
18	0.71 (0.25-2.01)		0.76 (0.27-2.13)	
≥ 20	2.51 (1.04-6.11)		2.58 (1.06-6.27)	
Linear (for each size up)	1.10 (0.98-1.25)	0.074	1.12 (1.00-1.25)	0.068

Fully adjusted model [1]: adjusted for age, smoking, education and physical activity level. This analysis excluded participants who were censored or were diagnosed with diabetes at enrolment.

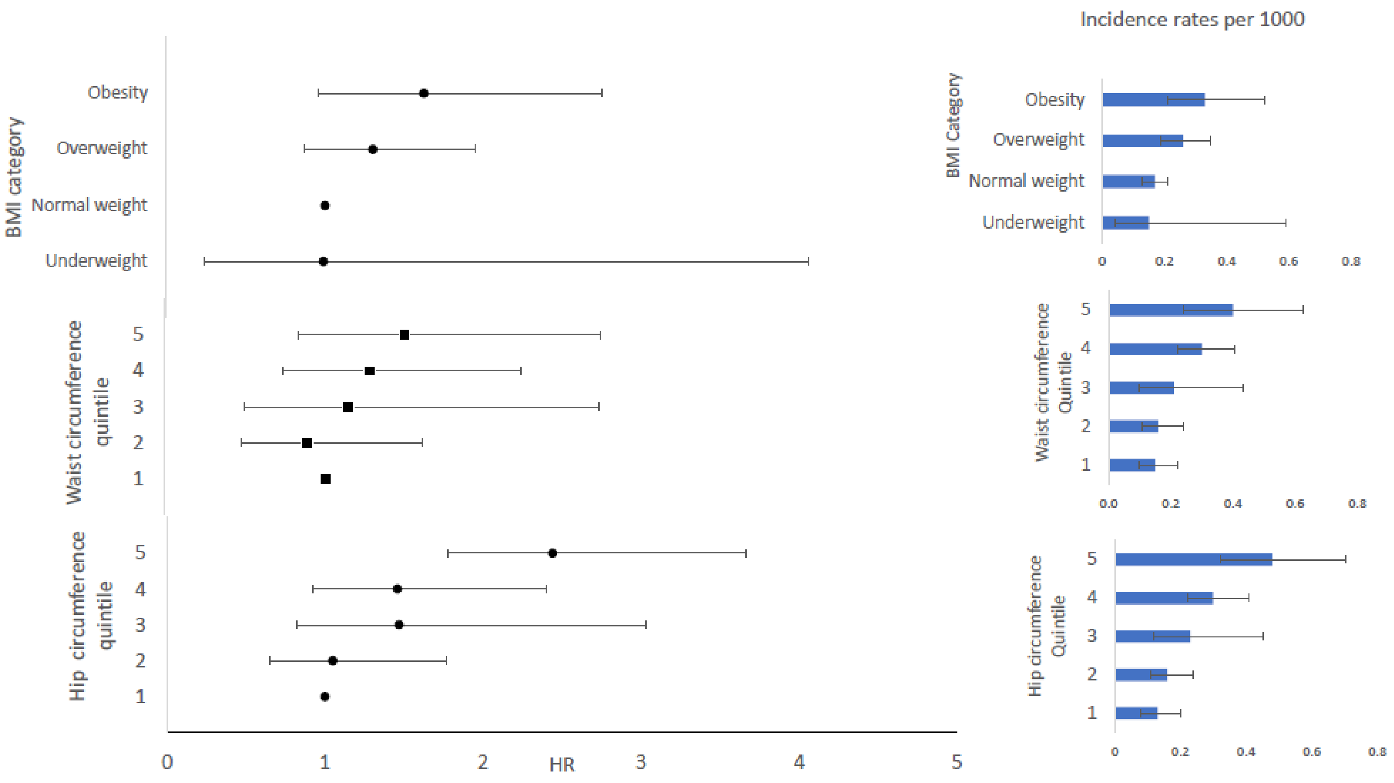

Figure S1. Fully adjusted HR (95%CI)and crude incidence rates for pancreatic cancer,by anthropometric indices. *Quintiles arranged in increasing order of circumference values.*

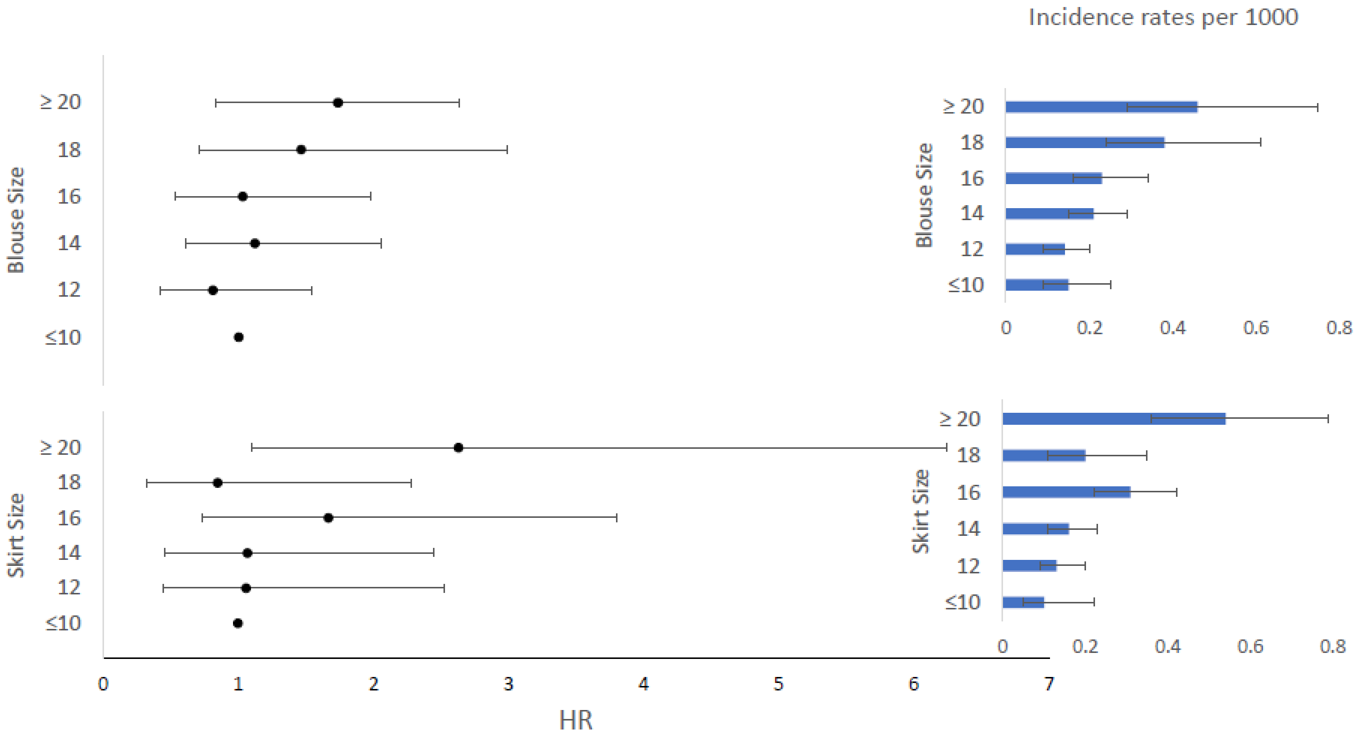

Figure S2. Fully adjusted HR (95% CI) and crude incidence rates for pancreatic cancer, by clothing size.
